# The Hospital Sunday Fund

**Published:** 1891-12-26

**Authors:** 


### PASSING TOPICS.

### THE HOSPITAL SUNDAY FUND.

THE report of the Council of the Hospital Sunday Fund is an interesting publication, and not a little may be learned by an attentive perusal of it. We are not disposed to say with the Lord Mayor that the success of the Fund has been "undoubtedly great," because it is only doing its promoters justice to assert that its success has not been in any way proportionate to its merits. His Lordship's promise of hearty co-operation is more to the point, and we sincerely hope that his year of office may be rendered memorable by, among other events, something like an awakening of the public conscience in regard to the value and wants of our great medical charities.

The Council are able to acknowledge receipts from all sources to a total of £45,330, being an increase of some £2,500 over the figures of last year, but included in the larger amount are two donations of £5,000 and £1,000 from the Duke of Cleveland and Sir Savile Crossley, respectively. Now, if exceptions can be taken to prove a rule, as the saying goes, do not the rotund and robust figures of these two notable contributions stand out in eloquent rebuke of the lean starvelings by which they are surrounded? Nearly one-seventh of the whole aggregate of a sum representing the offerings of five millions of souls is supplied by two men, neither of whom, to make the matter more striking, could be claimed as a regular Londoner.

Precisely the same lesson is taught by the fact that year by year one or two particular churches outstrip all others in respect of the sums collected. The number of contributing congregations is no less than 1,711, and two of them together yield as much as a sixteenth part of the total contributions. We are not among those who believe that any large and appreciable number of subscriptions to individual hospitals have been withdrawn in order that they may be replaced by a meagre contribution to the Sunday Fund. Certain persons may have been guilty of the meanness suggested, but we regard the Fund as actually no less than potentially an auxiliary to the ordinary subscription list. It is, however, more than possible that a large proportion of the total collection is contributed by those who are already subscribers. This cannot well be proved or disproved to demonstration, but it is only reasonable to believe that the majority of the supporters of our philanthropic institutions would be found naturally among the regular attendants at places of worship. If this be not so, we must be prepared to admit the truth of the charge that religion has little to do with humanity, and in that case what are we to think of the power of the Church to interpret the very foremost precept of Christianity?

If only all the clergy would but warm to the subject, and put forth their best powers in the way, for example, the Rev. Prebendary Forrest, the Rev. Canon Fleming, and the Rev. C. J. Ridgeway do, there would no longer be any reason that the Metropolitan Hospital Sunday Fund should be regarded by its best friends with somewhat mixed feelings. It is an institution gifted with splendid opportunities, but hitherto it cannot be said to have made full use of them. It is a case with which a great deal of zeal will not be amiss.

AGRICOLA.

---

COOK your water as you do other food is certainly sound advice, especially if there be added to it the advice to treat your milk in the same way. But the suggestion, though repeatedly given when typhoid and other epidemics break out, is seldom acted upon. The objection generally brought against boiled milk and water is that they are so unpalatable. This is not necessarily the case; water if well filtered after being boiled becomes perfectly palatable; if, however, it is not filtered, it is flat and unpleasant to the palate.

